# Molecular Characterization and Phylogenetic Analysis of *Enterocytozoon bieneusi* in Lambs in Oromia Special Zone, Central Ethiopia

**DOI:** 10.3389/fvets.2020.00006

**Published:** 2020-01-29

**Authors:** Teklu Wegayehu, Junqiang Li, Md. Robiul Karim, Longxian Zhang

**Affiliations:** ^1^College of Natural Sciences, Arba Minch University, Arba Minch, Ethiopia; ^2^College of Animal Sciences and Veterinary Medicine, Henan Agricultural University, Zhengzhou, China; ^3^Scientific Research Experiment Center & Laboratory Animal Center, Henan University of Chinese Medicine, Zhengzhou, China; ^4^Faculty of Veterinary Medicine and Animal Science, Bangabandhu Sheikh Mujibur Rahman Agricultural University, Gazipur, Bangladesh

**Keywords:** *Enterocytozoon bieneusi*, genotypes, internal transcribed spacer, lambs, Ethiopia

## Abstract

*Enterocytozoon bieneusi* is the most frequently diagnosed microsporidian species in humans and occurs in a wide range of animals. This study was conducted in Central Ethiopia to determine the prevalence and genotypes of *E. bieneusi* in lambs in order to evaluate their public health significance. Three hundred eighty nine fecal samples were collected and screened using a nested PCR targeting the internal transcribed spacer (ITS) of the ribosomal RNA gene. All positive PCR products were sequenced to determine the genotypes. *E. bieneusi* was found in 39 (10.03%) of the lambs. Differences in the infection rates among sex and age groups were not significant (*P* > 0.05). Five ITS genotypes belonging to three known genotypes BEB6, COS-I, and COS-II, and two novel genotypes (ET-L1 and ET-L2) were identified in lambs. All five genotypes identified in the present study clustered within cattle-specific Group 2 in the ITS phylogenetic tree. This first molecular detection and characterization of *E. bieneusi* in lambs in Ethiopia has identified the need for further studies in humans and other domestic animals in order to determine the public health significance of *E. bieneusi* in Ethiopia.

## Introduction

Microsporidia are obligate intracellular pathogens in humans and other animals worldwide (1, 2). Among the four microsporidian species infecting humans, *Enterocytozoon bieneusi* is the most frequently diagnosed species. It has also been reported in many mammals and birds (1). *E. bieneusi* primarily infects the intestinal absorptive cells and is mainly associated with chronic diarrhea and wasting syndrome (3).

Based on the internal transcribed spacer (ITS) nucleotide sequence, considerable genetic diversity has been found within *E. bieneusi* (1, 4). To date, more than 500 genotypes of *E. bieneusi* have been reported (5). The findings of previous studies suggest that both host-adapted genotypes with narrow host ranges and potentially zoonotic *E. bieneusi* genotypes with wide host ranges have been identified (1, 6).

The presence of 11 genetic groups (Group 1 to 11) has been described by phylogenetic analysis of *E. bieneusi* ITS sequences (5). Group 1 is the largest group containing 314 genotypes, which are commonly found both in humans and animals and considered to be zoonotic (5). The second largest group, Group 2, was considered to be adapted to ruminants (7). However, the frequent observation of some Group 2 genotypes, such as BEB4, BEB6, I, and J in other animals and humans suspends their host specificity and implies the zoonotic potential of some genotypes in this group. The genotypes in remaining genetic Groups 3–11 appear to be more host-specific and probably have no public health significance (5).

Aside from two studies in humans using microscopy and Polymerase Chain Reaction (PCR), *E. bienusi* has not been studied and characterized in Ethiopia (8, 9). The aim of this study was to determine the prevalence and the infecting genotypes of *E. bieneusi* in lambs in Oromia Special Zone, Central Ethiopia to extrapolate the public health importance.

## Materials and Methods

### Study Area

**Figure 1:**
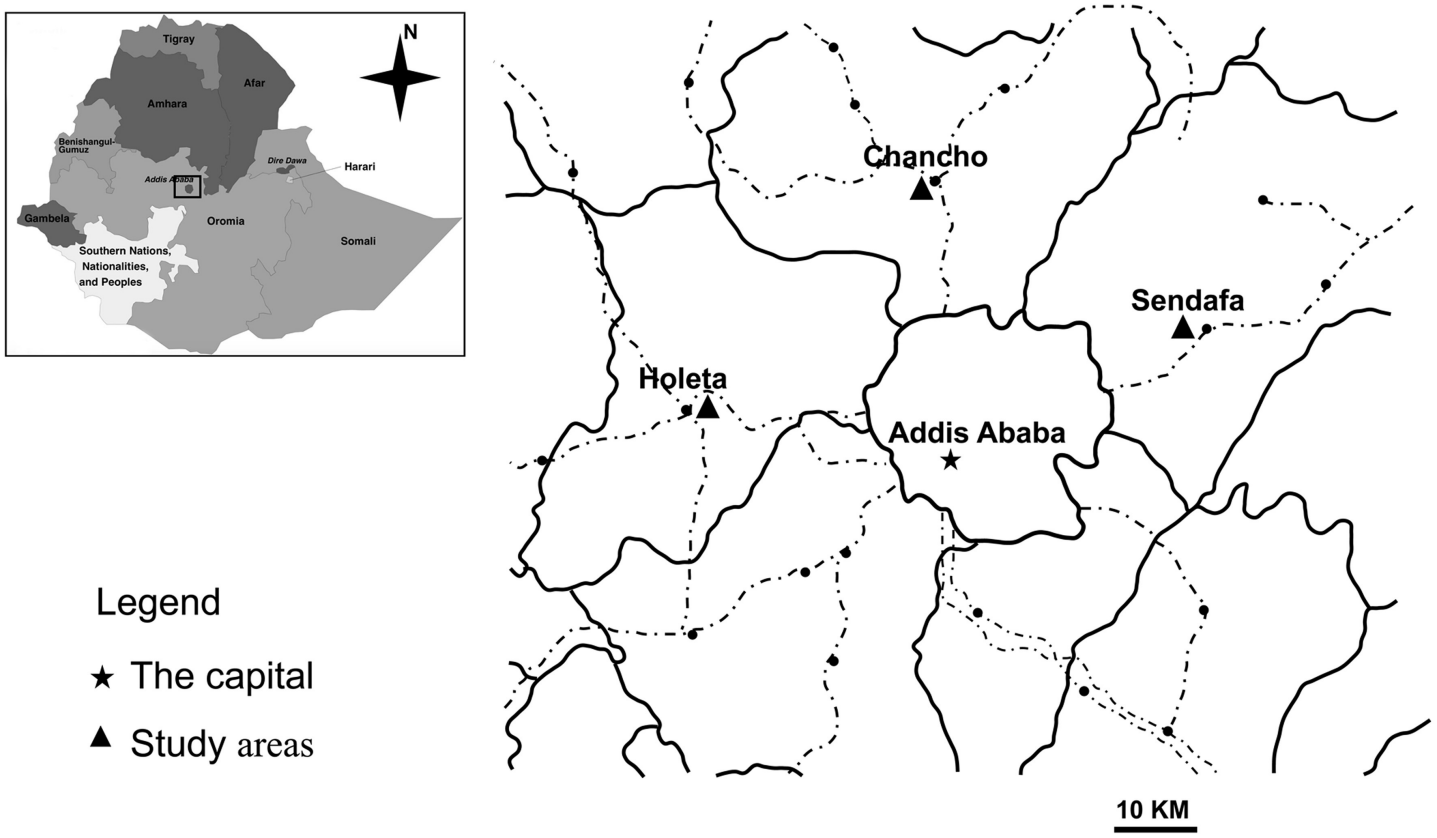
Locations of the study area in Oromia Special Zone, Central Ethiopia [Adapted from Wegayehu et al. (10)].

This study was conducted in Oromia Special Zone, Central Ethiopia. It is one of the zones in Oromia Region and bordered with Eastern Shewa zone in the east, North Shewa zone in the North East and South-west Shewa Zone in the South West. The mean annual temperature of the Zone is found between 20 and 25°C in the lowlands and 10–15°C in the central highlands. Based on the available meteorologically data, the mean annual rainfall varies from 700 to 1,400 mm in lowlands and highlands, respectively. Of the eight major towns of the Special Zone, Holeta, Sendafa, and Chancho towns were included in this study because of high sheep density (Figure 1).

### Sample Animal and Specimen Collection

The fecal samples were collected from lambs younger than 3 months. The owners of the lambs who consent to provide fecal samples were included in this study. A total of 389 fresh fecal samples were collected from lambs in separate and labeled stool containers. The specimens were taken directly from the rectum of each animal or immediately after defecation using sterile disposable gloves. Identification number, animal species, age, and sex were recorded during sample collection. The specimens were preserved in 2.5% potassium dichromate solution, transported immediately to the laboratory on ice and stored at 4°C prior to deoxyribonucleic acid (DNA) extraction.

### DNA Extraction

The preserved fecal specimens were washed with deionized water to remove the preservative. Genomic DNA was extracted from each fecal sample using the E.Z.N.A.® Stool DNA kit (Omega Biotek Inc., Norcross, USA). Briefly, about 200 mg of fecal specimen was added in a 2 ml centrifuge tube containing 200 mg of glass beads and placed on ice. Following, 300 μl buffer SP1 and proteinase K were added, and incubated at 70°C for 10 min. Subsequently, all the procedures outlined in product manual were performed according to the manufacturer's protocol. Finally DNA was eluted in 200 μl of elution buffer and the extract was stored at −20°C until used in PCR.

### PCR and Sequence Analysis

*Enterocytozoon bieneusi* was detected using nested PCR targeting the ITS region of the rRNA gene (6). The first-round PCR was performed in a 25 μl reaction volume that includes 23 μl mixes (TaKaRa Bio, Otsu, Japan) and 2 μl DNA template. The primary PCR program was involved enzyme activation at 94°C for 5 min, followed by denaturation at 94°C for 30 s, annealing at 57°C for 30 s and primer extension at 72°C for 40 s for 35 cycles. A 7 min final extension at 72°C was done after 35 cycles followed by cooling at 4°C. The second-round PCR was conducted in a 25 μl reaction volume consisting of 23 μl mixes (TaKaRa Bio, Otsu, Japan) and 2 μl product of first PCR. The second PCR was run under the same conditions as the first except the annealing temperature which was reduced from 57 to 55°C for 30 s; and the cycle which was again reduced from 35 to 30 cycles. The amplificons were separated by electrophoresis on 1% agarose gel and visualized under a trans-illuminator after staining with ethidium bromide. To avoid contamination in PCR amplification, several precautions were taken, such as changing gloves prior to handling positive controls, using a separate set of pipettes and pipetting PCR reagents and amplified products in separate areas.

The secondary PCR products were purified using Montage PCR filters (Millipore, Bedford, MA) and sequenced using an ABI BigDye Terminator v. 3.1 cycle sequencing kit (Applied Biosystems, Foster City, CA) on an ABI 3100 automated sequencer (Applied Biosystems). The nucleotide sequences obtained were aligned with reference *E. bieneusi* sequences using ClustalX software (ftp://ftp-igbmc.u-strasbg.fr/pub/ClustalX/). The established nomenclature system was used in naming novel *E. bieneusi* ITS genotypes (4).

Bayesian inference (BI) and Monte Carlo Markov chain methods were used to construct the phylogenetic trees in MrBayes program (version 3.2.6). The posterior probability values were calculated by running 1,000,000 generations. A 50% majority rule consensus tree was constructed from the final 75% of the trees generated via BI. Analyses were run three times to ensure convergence and intensity to priors.

### Data Analysis and Nucleotide Sequence Accession Numbers

Data were entered into computer using EpiData version 3.1 and transferred to STATA Software for analysis. Chi square test was used to verify association of *E. bieneusi* infections in different study groups. *P*-values were considered to be statistically significant when <0.05. The representative nucleotide sequences of the five *E. bieneusi* ITS genotypes obtained in the present study were deposited in the GenBank database under the accession numbers: KT948316, KT948317, and MN728943 to MN728945.

## Results

### *E. bieneusi* Infection in Lambs

Of the 389 fecal specimens screened by nested ITS-PCR (392 bp) of the rRNA gene, *E. bieneusi* was found in 39 (10.03%) lambs (Table 1). The prevalence of *E. bieneusi* varied across the study areas. Relatively higher prevalence (15.53%, 16/103) was found in Holeta followed by Chancho (9.38%, 12/128) and Sendafa (6.96%, 11/158). The prevalence difference was statistically not significant (χ^2^ = 5.1686, *P* = 0.075) among the three study areas.

**Table 1:** Prevalence of *E. bieneusi* in lambs by study areas, sex, and age in Oromia Special Zone, central Ethiopia (January–June, 2014).

**Demographic characteristics**	**No. of samples examined**	**No. of samples positives (%)**	**Genotypes (no. of specimens)**
**STUDY AREAS**			
Holeta	103	16 (15.53)	BEB6 (6), COS-I (2), COS-II (1), ET-L1 (3), ET-L2 (4)
Sendafa	158	11 (6.96)	BEB6 (4), COS-I (3), ET-L1 (2), ET-L2 (2)
Chancho	128	12 (9.38)	BEB6 (3), COS-I (2), ET-L1 (3), ET-L2 (4)
**SEX**			
Male	188	17 (9.04)	BEB6 (5), COS-I (5), ET-L1 (4), ET-L2 (3)
Female	201	22 (10.95)	BEB6 (8), COS-I (2), COS-II (1), ET-L1 (4), ET-L2 (7)
**AGE GROUPS**			
<5 weeks	90	6 (6.67)	BEB6 (2), ET-L1 (1), ET-L2 (3)
5–8weeks	163	20 (12.30)	BEB6 (8), COS-I (3), COS-II (1), ET-L1 (4), ET-L2 (4)
>8 weeks	136	13 (0.96)	BEB6 (3), COS-I (4), ET-L1 (3), ET-L2 (3)
**Total**	**389**	**39 (10.03)**	BEB6 (13), COS-I (7), COS-II (1), ET-L1 (8), ET-L2 (10)

#### *E. bieneusi* Infection by Sex, Age, and Breed Groups

To investigate the distribution of *E. bieneusi* among lambs in relation to sex and age groups, data were arranged and summarized in Table 1. The prevalence of *E. bieneusi* was 9.04% (17/188) in male and 10.95% (22/201) in female lambs which was statistically not significant between the two groups. Although the age-associated difference in the occurrence of *E. bieneusi* infections was statistically not significant (χ^2^ = 0.3899, *P* = 0.532), lambs aged between 5 and 8 weeks had the highest infection rate with 12.30% (20/163) (χ^2^ = 2.029, *P* = 0.358).

#### Genotypes of *E. bieneusi*

The sequence analysis of 39 positive lamb specimens revealed the presence of five different genotypes in lambs. Three of them (BEB6, COS-I, and COS-II) were known genotypes and two (designated as ET-L1 andET-L2) were novel. The prevalent genotype was BEB6, being observed in 13 specimens followed by ET-L2, ET-L1, and COS-I observed in 10, 8, and 7 specimens, respectively. Genotype COS-II was found only in one specimen (Table 1).

### Phylogenetic Analysis

**Figure 2:**
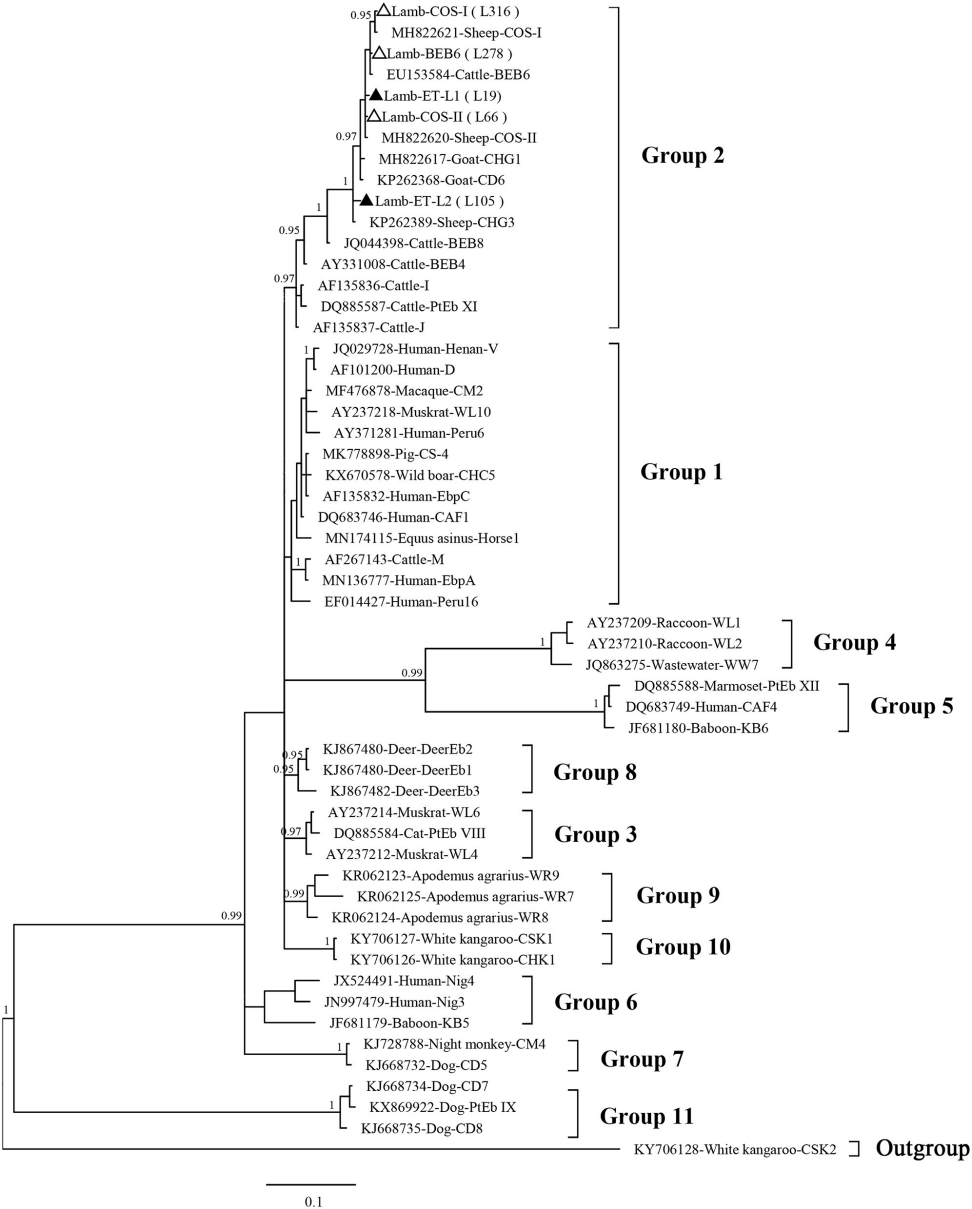
Phylogenetic tree based on Bayesian inference (BI) analysis of the *Enterocytozoon bieneusi* ITS sequences. Statistically significant posterior probabilities (0.95 and higher) are indicated on the branches. Known and novel *E. bieneusi* ITS genotypes identified in the present study are indicated by empty and filled triangles, respectively.

Phylogenetic analysis revealed that the three known (BEB6, COS-I, and COS-II) and two novel (ET-L1 and ET-L2) genotypes identified herein all were clustered into the Group 2 (Figure 2).

## Discussion

*Enterocytozoon bieneusi* has been the focus of numerous epidemiological studies both in humans and animals because of its opportunistic and zoonotic potential. However, there is knowledge gap on *E. bienusi* epidemiology in Ethiopia. So far, this parasite has been studied and characterized in a few human samples in the country (8, 9). This is the first investigation of *E. bieneusi* in animals in Ethiopia.

In the present study, *E. bieneusi* was found in 10.03% of lambs in Central Ethiopia. Similar rate of infection was reported for *E. bieneusi* in sheep in Northeast China (13.9%, 68/489) (11), Southwest China (10.6%, 70/661) (12), and Egypt (11.2%, 10/89) (13); while lower infection rates were detected in sheep in East-central China (3.4%, 28/832) (14) and Slovakia (0/33) (15). However, a higher prevalence of *E. bieneusi* was found in Inner Mongolian (China) lambs (77.8%, 126/162) (16), Swedish lambs (68%, 49/72) (17), Brazilian sheep (19.2%, 24/125) (18), and in sheep from some other studies in China (19–22). The differences in the prevalence are likely due to differences in the geographic and ecological setup.

Although not significant, *E. bieneusi* infection was slightly higher in female lambs than males. This is in accordance with report from China (19), who showed somewhat higher prevalence in female sheep. Together, these findings suggest that infection occurs regardless of the sex of the lambs.

Many studies showed the variation in infection rates of *E. bieneusi* in different age group of sheep, as well as in lambs (12, 14, 17, 21). In present study, only lambs younger than 3 months were assessed. Although, statistically not significant, lambs aged between 5 and 8 weeks had the highest infection rate (12.3%).

The genotype BEB6 identified in cattle in the United States (23), was also identified as the dominant *E. bieneusi* genotype (13/39, 33.3%) in lambs in the present study. Many other studies demonstrated that the genotype BEB6 was the dominant *E. Bieneusi* genotype in sheep in Sweden (17), Brazil (18), and China (16, 21). Although this genotype was considered to be cattle-specific, it is now recognized as a dominant genotype in humans, animals and birds in a wide geographic distribution, and has zoonotic potential (11, 17, 19, 24, 25). Genotypes COS-I and COS-II, commonly reported in cattle, sheep, deer, and non-human primates in previous studies (2, 14, 26, 27), were recognized as the known *E. bieneusi* genotypes. In contrast, the novel genotypes (ET-L1 and ET-L2) were first identified in lambs in this study. The sequence of new genotype ET-L1 (KT948316) had one nucleotide difference (G-to-T substitution) from that of genotype COS-II (KJ850433) at position 265, whereas sequence of new genotype ET-L2 (KT948317) had one nucleotide difference (G-to-A substitution) from that of genotype CM21 (KU604931) at position 174.

The phylogenetic analysis revealed that all five genotypes identified herein are clustered into the so-called cattle-specific Group 2. On other hand, no genotype was clustered under the zoonotic Group 1. However, some genotypes in the Group 2, such as BEB6, I, and J, have been detected in humans in recent years and are a potential source of zoonotic transmission between sheep or other animals and humans (2, 28).

The previous studies conducted in patients infected with the human immunodeficiency virus-1 in Ethiopia simply showed the prevalence of *E. bieneusi* infection using PCR and Uvitex-2B stain (8, 9). As ITS-genotyping was not performed it is not clear which genotype was infecting humans. Further molecular studies are needed to understand the patterns of transmission.

## Conclusions

This is first molecular investigation of *E. bieneusi* in Ethiopia, revealed that this species is the most prevalent microsporidia in lambs. Five *E. bieneusi* genotypes (three known and two novel) were identified in lambs in present study, and genotype BEB6 was the dominant genotype. Further studies in humans and other domestic animals are necessary to assess the public health significance of *E. bieneusi* in Ethiopia.

## Data Availability Statement

The datasets generated for this study can be found in the Nucleotide sequences of the ITS of the two novel *E. bieneusi* genotypes obtained in the present study (ET-L1 and ET-L2) were deposited in the GenBank database under accession numbers KT948316 and KT948317.

## Ethics Statement

This animal study was reviewed and approved by National Health Research Ethics Review Committee, Ministry of Science, and Technology. Support letters were obtained from concerned health and agricultural offices and administrative authorities at community level. Written informed consent was obtained from the owners for the participation of their animals in this study.

## Author Contributions

TW and LZ conceived the idea. TW, JL, and MK conducted the experiments. TW, JL, MK, and LZ wrote and revised the manuscript.

### Conflict of Interest

The authors declare that the research was conducted in the absence of any commercial or financial relationships that could be construed as a potential conflict of interest.
